# Cranberry Arabino-Xyloglucan and Pectic Oligosaccharides Induce *Lactobacillus* Growth and Short-Chain Fatty Acid Production

**DOI:** 10.3390/microorganisms10071346

**Published:** 2022-07-03

**Authors:** Arland T. Hotchkiss, John A. Renye, Andre K. White, Alberto Nunez, Giselle K. P. Guron, Hoa Chau, Stefanie Simon, Carlos Poveda, Gemma Walton, Robert Rastall, Christina Khoo

**Affiliations:** 1 Dairy & Functional Foods Research Unit, Agricultural Research Service, United States Department of Agriculture, Wyndmoor, PA 19038, USA; john.renye@usda.gov (J.A.R.J.); andre.white@usda.gov (A.K.W.); alberto.nunez@usda.gov (A.N.); giselle.guron@usda.gov (G.K.P.G.); rose.chau@usda.gov (H.C.); 2 Sustainable Biofuels and Co-Products Research Unit, Agricultural Research Service, United States Department of Agriculture, Wyndmoor, PA 19038, USA; stefanie.simon@usda.gov; 3 Department of Food and Nutritional Sciences, The University of Reading, Harry Nursten Building, Pepper Lane, Whiteknights, Reading RG6 6DZ, UK; c.g.povedaturrado@reading.ac.uk (C.P.); g.e.walton@reading.ac.uk (G.W.); r.a.rastall@reading.ac.uk (R.R.); 4 Ocean Spray Cranberries, Inc., One Ocean Spray Drive, Lakeville-Middleboro, MA 02349, USA; ckhoo@oceanspray.com

**Keywords:** cranberry, xyloglucan, rhamnogalacturonan, arabinogalactan, prebiotic, *Lactobacillus*, short-chain fatty acids

## Abstract

Numerous health benefits have been reported from the consumption of cranberry-derived products, and recent studies have identified bioactive polysaccharides and oligosaccharides from cranberry pomace. This study aimed to further characterize xyloglucan and pectic oligosaccharide structures from pectinase-treated cranberry pomace and measure the growth and short-chain fatty acid production of 86 *Lactobacillus* strains using a cranberry oligosaccharide fraction as the carbon source. In addition to arabino-xyloglucan structures, cranberry oligosaccharides included pectic rhamnogalacturonan I which was methyl-esterified, acetylated and contained arabino-galacto-oligosaccharide side chains and a 4,5-unsaturated function at the non-reducing end. When grown on cranberry oligosaccharides, ten *Lactobacillus* strains reached a final culture density (ΔOD) ≥ 0.50 after 24 h incubation at 32 °C, which was comparable to *L. plantarum* ATCC BAA 793. All strains produced lactic, acetic, and propionic acids, and all but three strains produced butyric acid. This study demonstrated that the ability to metabolize cranberry oligosaccharides is *Lactobacillus* strain specific, with some strains having the potential to be probiotics, and for the first time showed these ten strains were capable of growth on this carbon source. The novel cranberry pectic and arabino-xyloglucan oligosaccharide structures reported here combined with the *Lactobacillus* strains that can metabolize cranberry oligosaccharides and produce short-chain fatty acids, have excellent potential as health-promoting synbiotics.

## 1. Introduction

Consumption of foods containing sufficient amounts of dietary fiber has been associated with a wide variety of health benefits, including the alleviation of gastrointestinal diseases [1]. Dietary fiber includes oligosaccharides and polysaccharides that are classified as water insoluble or water soluble, and are most abundant in plant-based foods [2]. Cranberries are one such food ingredient that contains bioactive proanthocyanidins and oligosaccharides such as hemicellulose arabino-xyloglucan [3,4]. Consumption of cranberry juice has long been used to prevent urinary tract bacterial infections with proanthocyanidins thought to be responsible for this anti-adhesive activity [5]. However, several meta-analyses of clinical studies have reported conflicting results following consumption of proanthocyanidins from cranberry juice or tablets [6,7,8]. Therefore, other cranberry compounds may also play a role in prevention of urinary tract infections [3,4,9,10].

Cranberry oligosaccharides are one of those compounds that have emerging evidence for their bioactivity against uropathogenic strains of *E. coli*. Cranberry arabino-xyloglucan oligosaccharides with SSGG structure (arabino-xylo- side chains) blocked the adhesion of *E. coli* O157:H7 to colon HT29 cells and *E. coli* CTF073 and UT189 to urinary tract T24 cells [3]. The cranberry arabino-xyloglucan oligosaccharides had the highest affinity for *E. coli* with type 1 fimbriae [3]. Cranberry xyloglucan, arabinan and homogalacturonan oligosaccharides inhibited biofilm formation and quiescence by urinary tract and oral pathogens [11,12,13]. Carrot oligogalacturonic acids with a degree of polymerization (DP) of two and three were reported to have antiadhesive activity against *E. coli* binding to uroepithelial cells [14].

The cranberry pectic oligosaccharide structures suggested that cranberry oligosaccharides might also have prebiotic properties since arabino-oligosaccharides are well-known for their in vitro prebiotic positive modulation of the microbiota [15,16,17,18,19,20,21,22,23,24,25]. *Lactobacillus* spp. and *Bifidobacterium* spp. are commonly used probiotics, and when delivered with a prebiotic fiber, the resulting synbiotic enhances the probiotic survival. For example, the prebiotic raffinose increased the viability of the probiotic *Lactobacillus casei* ATCC 393 within yogurt [26]; and the inclusion of fructo-oligosaccharides within a cheese containing the probiotic *L. paracasei* INIA P272 improved its survival within the consuming host [27]. However, only specific strains among these genera can utilize prebiotics for growth. Strain specificity has been reported for strains of *Lactobacillus* spp. and *Bifidobacterium* spp. for growth on human milk oligosaccharides [28], and the utilization of commercial galacto-oligosaccharides for survival and colonization in the gastrointestinal tract [29]. Additionally, cranberry arabino-xyloglucan oligosaccharides were reported to support the growth of *Lactobacillus plantarum* BAA 793 and *Bifidobacterium longum* UCD401, although *L. johnsonii* ATCC 33200 and several strains of *B. longum* and *B. infantis* were unable to utilize these oligosaccharides [30]. Additionally, another study reported that while the metabolic pathways required for utilization of complex oligosaccharides in *Lactobacillus* spp. have been characterized [31], including the roles of α-L-arabinofuranosidase, β-xylosidase and exo-oligoxylanase for xyloglucan utilization [32], more work is needed to fully understand the strain specificity observed with prebiotic utilization by lactobacilli.

A primary benefit of enriching for bacteria able to metabolize oligosaccharides is the production of short chain fatty acids (SCFA) such as acetic, propionic, and butyric acids [33]. SCFA produced in the human colon have been associated with improved host metabolism and immunity [33,34]. Greater amounts of acetate, formate and less lactate were produced when *Lactobacillus plantarum* BAA 793 and *Bifidobacterium longum* UCD401 were grown in the presence of arabino-xyloglucan oligosaccharides [30]. Additionally, increased production of SCFA has been observed when xyloglucans were added to a batch fecal fermentation compared to the control samples, highlighting the potential benefits of xyloglucans as a functional food ingredient [35].

There is a need to further understand how certain strains are capable of producing SCFA and what constituents are necessary for their production. Arabino-xyloglucan structures were identified from a pectinase-treated cranberry pomace fraction (A6) [3]. Here, we used preparative HPLC to further purify cranberry A6 and characterized additional pectic and arabino-xyloglucan structures. An arabino-xyloglucan-enriched preparative HPLC fraction was used to further identify bacterial strains that can utilize cranberry oligosaccharides and to measure the resulting SCFA production by lactic acid bacteria (LAB) among intestinal microbiota, through fecal batch fermentation. Results from this study could identify potential synbiotics comprising a probiotic LAB strain and cranberry oligosaccharides.

## 2. Materials and Methods

### 2.1. Materials

The cranberry A6 fraction was produced as described previously [3]. Briefly, a cranberry (harvested in Massachusetts, USA 2008–2015) hull fraction (A1) was produced using Klerzyme 150 pectinase (DSM Food Specialties, South Bend, IN, USA) during the depectinization step of commercial cranberry juice processing. Fractionation of A1 was by reversed phase chromatography using a FLASH-40 system (Biotage, Charlotte, NC, USA) and a Biotage SNAP KP-C18-HS 120 g cartridge. The 15% methanol/water fractions were combined and dried to produce an A2 fraction. The A2 was further purified by Sephadex LH20 (Sigma-Aldrich, St. Louis, MO, USA) chromatography with deionized water elution followed by lyophilization to produce the A6 fraction. Batches of cranberry A6 produced in different years were reported as A6 A, A6 B and A6 C.

### 2.2. High Performance Size Exclusion Chromatography (HPSEC)

Dried cranberry A6 was dissolved in the mobile phase (0.05 mol·L^−^^1^ NaNO3/0.01% NaN3) at 5 mg/mL and filtered through a 0.45 μm Millex HV filter (Merck Ltd., Tullagreen, Ireland) before HPSEC analysis. The HPSEC columns consisted of three TSK GMPWxl, 7.8 × 300 mm, 13 μm particle size exclusion columns (Tosoh Bioscience, Tokyo, Japan) in series. Guard column PWXL with dimensions 6.0 mm I.D. × 4.0 cm, pore size 12 μm were placed directly before and after the column set (Tosoh Bioscience). The mobile phase used was 0.05 mol·L^−1^ NaNO3/0.01% NaN3 at a flow rate of 0.7 mL/min. The columns were heated in water bath set at 35 °C. The HPLC system was comprised of a model 1200 series degasser, auto sampler and solvent pump (Agilent Technology, Santa Clara, CA, USA). The detectors connected in series were a UV-1260 Infinity spectrophotometer (UV/VIS) (Agilent), HELEOS II multi-angle laser light scattering photometer (MALLS) (Wyatt Technology, Santa Barbara, CA, USA), model 255- V2 differential pressure viscometer (DP) (Wyatt Technology), and a differential refractive index detector (dRI) (Wyatt Technology). All detector signals were sent to a desktop computer for processing with ASTRA (Ver. 7.1.2.5) software (Wyatt Technology). The specific refractive index increment (dn/dc) describes the change in refractive index of a solution with the change of the polymer concentration. The dn/dc value used for cranberry was 0.132 and samples were analyzed in triplicate.

### 2.3. Monosaccharide Analysis

The monosaccharide analysis procedures were reported previously [36] with slight modifications. Methanolysis of samples was performed prior to high-performance anion-exchange chromatography with pulsed amperometric detection (HPAEC-PAD) using a CarboPac PA-20 column and guard column operated at 0.5 mL/min. Neutral and acidic monosaccharides were separated with a 11 mmol·L^−1^ NaOH isocratic (10 min) mobile phase, then a 0–90 mmol·L^−1^ sodium acetate (CH3COONa) gradient in 100 mmol·L^−1^ NaOH (30 min). The column was eluted with 1.0 mol·L^−1^ CH3COONa for 0.8 min before the mobile phase returned to 11 m mol·L^−1^ NaOH (30 min) prior to the next injection.

### 2.4. Oligosaccharide Structure

Oligosaccharide structure was determined by Matrix-Assisted Laser Desorption/Ionization Mass Spectrometry with a 4700 Proteomics Analyzer mass spectrometer (Sciex, Framingham, MA, USA) in the positive reflectron mode. Spectra were obtained by averaging 1000 acquired spectra in the MS mode. Conversion of TOF to mass (Da) for the monoisotopic ions, (M + Na)+ and (M + K)+, was based on calibration of the instrument with a peptide standard calibration kit (Sciex). Dried cranberry A6 was dissolved in 1 mL of water and cleaned with graphitized carbon tips (NuTip 10–200 µL, Glygen Co., Columbia, MD, USA). The tips were first conditioned by passing 100 mL of acetonitrile:water (70:50) and then washed 4 times with 100 µL of water. After conditioning, 100 µL of the oligosaccharide solution was loaded on the tip, washed 3 times with 100 µL of water and the oligosaccharides were eluted with 100 µL of acetonitrile:water (30:70) 0.1% trifluoroacetic acid (TFA) (*v*/*v*). The extracted solution was dried with a vacuum centrifuge, resuspended in 5 mL of a solution of 2,5-dihydroxy benzoic acid (10 mg/mL in acetonitrile:water (50:50) 0.1% TFA (*v*/*v*)), and spotted onto a MALDI plate for analysis (1 µL).

### 2.5. Oligosaccharide Glycosyl-Linkage Positions

Glycosyl linkage analysis was performed at the Complex Carbohydrate Research Center (University of Georgia, Athens, GA, USA) by combined gas chromatography/mass spectrometry (GC/MS) of the partially methylated alditol acetates (PMAAs) derivatives produced from cranberry A6. The procedure was a slight modification of the one described previously [37]. Briefly, methylation of the samples using dimsyl potassium base was performed. This was followed by acetylation of any unmethylated hydroxyl groups using N-methylimidazole and acetic anhydride. The samples were extracted with dichloromethane, and the carboxylic acid methyl esters were reduced using lithium aluminum deuteride in THF (80 °C, 8 h). After desalting using an On-guard H+ column (Thermo Fisher, Waltham, Massachusetts, MA, USA), the samples were remethylated using two rounds of treatment with sodium hydroxide (15 min each) and methyl iodide (45 min each). The samples were then hydrolyzed using 2 mol·L^−1^ TFA (2 h in sealed tube at 120 °C), reduced with NaBD4, and acetylated using acetic anhydride/TFA. The resulting PMAAs were analyzed on an Agilent 7890A GC interfaced to a 5975C MSD (mass selective detector, electron impact ionization mode); separation was performed on a 30 m Supelco SP-2331 bonded phase fused silica capillary column.

### 2.6. Preparative HPLC

Cranberry A6 was dissolved in deionized water to create a final concentration of 20 mg/mL. This solution was syringe-filtered (0.45 µm) into autosampler vials and injections were made on a 21.2 × 250 mm Rezex RSO-Oligosaccharide Ag+ 4% preparative column (Phenomenex, Torrance, CA, USA) with a particle size of 12 um and a Rezex Ag+ 15 × 21.2 mm ID guard cartridge (Phenomenex). The HPLC system also included an AS3500 Autosampler (Thermo Scientific,), a Model 303 gradient pump (Gilson, Inc., Middleton, WI, USA), an RID-10A refractive index detector (Shimadzu, Inc., Columbia, MD, USA), and an UltiMate 3000 fraction collector (Dionex, Sunnyvale, CA, USA). Deionized water was used as the mobile phase, with a constant flow rate of 1.0 mL/min. The column was heated to 80 °C. Injections of 200 µL were made onto the column every 75 min. Three fractions were collected based on the positions of the three most prominent peaks. Like fractions were combined, frozen, and lyophilized.

### 2.7. Lactic Acid Bacteria Growth on Cranberry Xyloglucans

*Lactobacillus* (86) and *Weissella* (2) strains (Supplemental Table S1) were maintained in de Man, Rogosa and Sharpe medium (MRS, Becton Dickinson Difco, Franklin Lakes, NJ, USA) at 32 °C; and *Bifidobacterium breve* 2141 and *Bifidobacterium longum* 3300 were maintained in MRS supplemented with 0.05% cysteine and grown under anaerobic conditions. A modified MRS medium (mMRS) without glucose (1.0% *w*/*v* proteose peptone No. 3; 1.0% *w*/*v* beef extract; 0.5% *w*/*v* yeast extract; 0.1% *w*/*v*; polysorbate 80; 0.2% *w*/*v* ammonium citrate; 0.5% *w*/*v* sodium acetate; 0.01% *w*/*v* magnesium sulfate; 0.005% *w*/*v* manganese sulfate; 0.2% *w*/*v* dipotassium phosphate) was prepared and used to assess bacterial growth with oligosaccharides supplied as the sole carbon source. Oligosaccharide substrates included cranberry A6 (Ocean Spray, Middleborough, MA, USA), fructo-oligosaccharide (FOS) (Raftilose P95; Beneo, Parsippany, NJ, USA) and inulin (Raftilose Synergy 1; Beneo, Parsippany, NJ, USA). Substrates were added to basal mMRS at 1% *w*/*v*, with resulting solutions filtered sterilized (0.22 µm) and stored at 4 °C.

Bacterial strains were grown overnight in MRS broth (5 mL) at 32 °C, and then washed twice and resuspended in 0.1% peptone water (5 mL). Cells were diluted 20-fold into 200 µL of mMRS containing 1% prebiotic (cranberry A6, FOS or inulin). Bacteria were grown at 32 °C in the presence of 10% Oxyrase (Oxyrase Inc., Mansfield, OH, USA) to establish an anaerobic environment. Bacterial growth was monitored for 24 h using a Cytation 5 multimode plate reader (BioTek Instruments Inc., Winooski, VT, USA), with absorbance (600 nm) readings collected hourly. Optical density data (OD_600nm_) is presented as the ΔOD_600_, with the growth level calculated by subtracting the initial OD_600_ reading from all subsequent measurements [38]. Data is the average value from two biological replicates (±standard deviation; SD).

For short-chain fatty acid analysis, *Lactobacillus* and *Bifidobacterium* strains were grown in 1 mL mMRS containing 1% cranberry A6 in the presence of 10% Oxyrase at 32 °C for 24 h. Cultures were centrifuged at 13,000× *g* for 10 min, and cell free supernatants (CFS) were filtered (0.22 µm) prior to analysis by high performance liquid chromatography (HPLC) [16]. Short-chain fatty acid analysis was performed on a 20 µL sample using an Aminex HPX-87H column with Micro-Guard Cation H guard cartridge (Bio-Rad, Hercules, CA, USA) and a RID-20A RI detector (Shimadzu Corp., Kyoto, Japan), both maintained at 40 °C. A 5 mmol·L^−1^ sulfuric acid mobile phase was used with a flow rate of 0.6 mL/min. Peaks corresponding to known concentrations of lactic, acetic, propionic, and butyric acids were used to calculate the concentration of each SCFA within CFS. Results are the average of three replicates ± SD.

### 2.8. Miniature Fecal Batch Cultures

Miniature batch cultures (10 mL working volume) were performed in triplicate for each substrate: inulin (Raftilose Synergy 1; Beneo, Parsippany, NJ, USA); cranberry pomace (Ocean Spray, Middleborough, MA), and cranberry preparative HPLC fraction 2 (extract 2) from cranberry A6. Fecal donors were two healthy males and one healthy female aged between 24 and 35 years, who had not received antibiotic treatment for at least 6 months prior to experimentation. A separate fecal donor was used for each run and there were three runs of the batch cultures in total. Sterile pH-controlled batch culture fermentation vessels were aseptically filled with 9 mL of sterile basal nutrient medium and sparged with O_2_-free N_2_ (15 mL/min) overnight to establish anaerobic conditions. The substrates to be tested were dissolved in the prepared medium to give a final concentration of 1% (*w*/*v*), and each vessel was inoculated with 1 mL of freshly prepared fecal slurry, prepared by homogenizing fresh human feces from one donor (10%, *w*/*v*) in phosphate-buffered saline. Additionally, a vessel without any substrate was used as negative control. Batch culture fermentations were incubated for 48 h with gentle stirring, and samples were collected at times 0 h, 24 h and 48 h. Fluorescence in situ hybridization (FISH) analysis was performed in conjunction with flow cytometry (Flow FISH) as described previously, each sample was analyzed once and the triplicate data were used to calculate the mean and standard deviation [39]. Short chain fatty acid analysis was performed using a previously described modified derivatization method, again, each sample was analyzed once and the triplicate data were used to calculate the mean and standard deviation [39].

### 2.9. Statistical Analysis

Statistical analyses were performed using SPSS for Windows, version 21 (IBM, New York, NY, USA). Univariate analysis of variance and Tukey’s posthoc test was used to determine significant changes in the Log_10_ bacterial counts and SCFA concentrations at inoculation and subsequent sampling points, and to compare differences in the effects of substrates at the same time point. Differences were considered significant when *p* < 0.05.

## 3. Results and Discussion

### 3.1. Composition and Characterization by HPSEC

**Figure 1:**
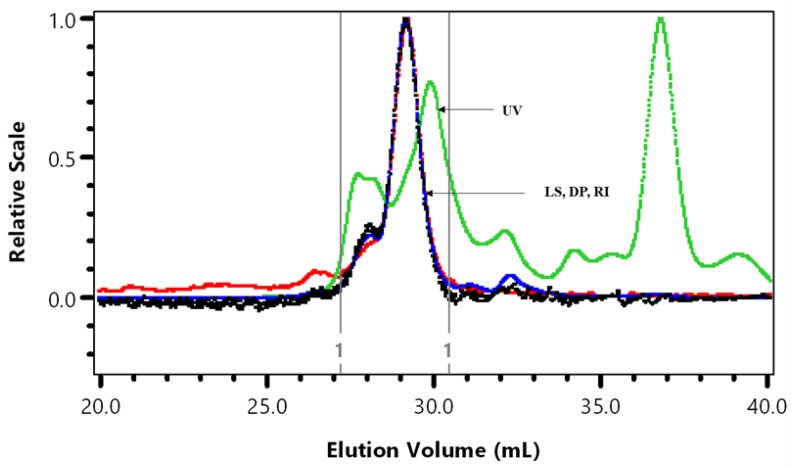
HPSEC superimposed UV at 280 nm, light scattering at 90° (LS), viscosity (DP) and refractive index (RI). The vertical lines are the integration by part limits of A6 cranberry fraction.

We previously published the weight average molar mass (Mw) of cranberry A6 as 2750 Da [3]. The HPSEC analysis of a more recent batch of A6 was repeated to demonstrate batch to batch variation (Figure 1, Table 1).

Some batch-to-batch variation in A6 average molar mass (*M_w_* × 10^−3^), intrinsic viscosity (*η_w_*), and hydrodynamic radius (Rhzv) was observed (Table 1). Sun et al. [11] predicted the average molecular size of 1370 Da for a cranberry A6 sample that was further purified using anion-exchange and gel filtration chromatography. The Mark-Houwink (M-H) value of 0.260 indicated a compact molecular shape in contrast to the random coil shape (0.701) observed for cranberry A6 in Hotchkiss et al. [3].

### 3.2. Monosaccharide and Oligosaccharide Composition

**Figure 2:**
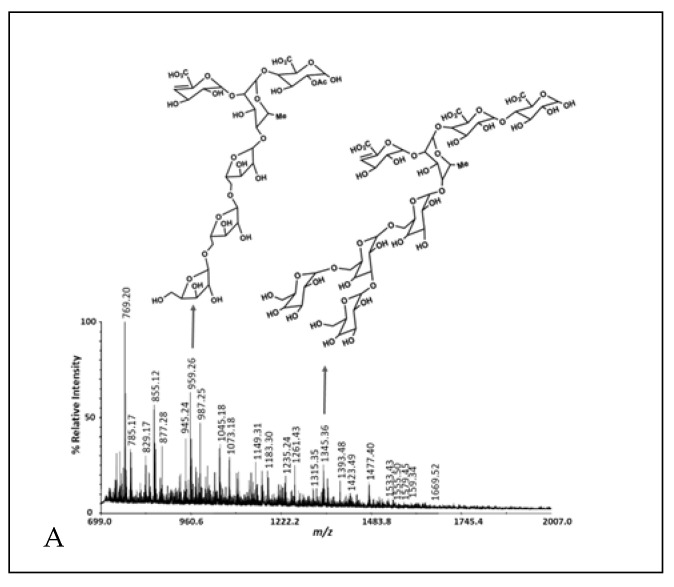

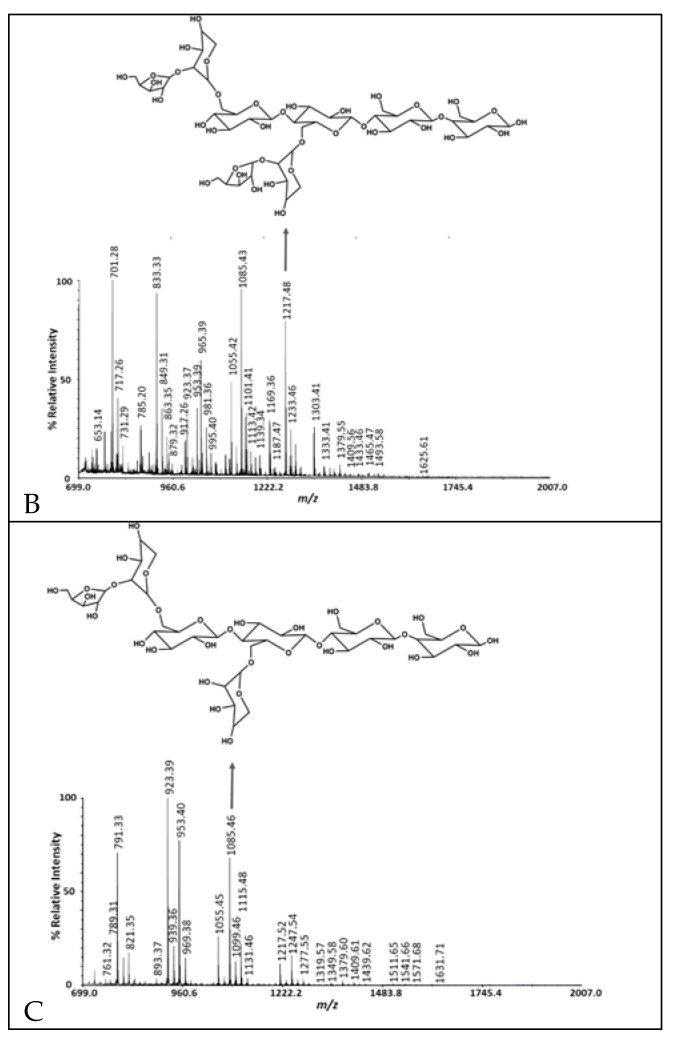
MALDI-TOF MS of cranberry A6 preparative HPLC fractions 1 (**A**), 2 (**B**) and 3 (**C**).

Including the A6 monosaccharide composition reported previously [3], the batch-to-batch range in monosaccharide mole % observed was glucose, 38.8–50.3%; arabinose, 16.1–25.7%; galactose, 0.9–4.4%; xylose, 6.8–30.4%; rhamnose, 0.5–31.9%; fucose, 0.1–0.4%; galacturonic acid, 1.0–7.9%; and glucuronic acid, 0.2–2.5% (Table 2). The cranberry A6 A batch was similar to the A6 monosaccharide composition reported previously [3], consisting of a combination of arabino-xyloglucan monosaccharides with minor amounts of pectic monosaccharides. Therefore, A6 A was used for the single strain growth and short-chain fatty acid production studies. We purified A6 into three preparative HPLC fractions using a Rezex RSO-Oligosaccharide Ag+ 4% column and a water mobile phase. Most of the galacturonic acid-containing oligosaccharides eluted in the first preparative HPLC fraction, while arabino-xyloglucan oligosaccharides eluted in the second and third fractions (Table 2, Figure 2). The pectic oligosaccharides in preparative HPLC fraction 1 had rhamnogalacturonan I structures that were methyl-esterified, acetylated with arabino- and galacto-oligosaccharide side chains and a 4,5-unsaturated function at their non-reducing ends (Figure 2, Supplementary Table S1). We reported cranberry pectic oligosaccharide structures previously [3] and further describe cranberry rhamnogalacturonan structures here (Figure 2A, Supplementary Table S1). The cranberry arabino-xyloglucan structures in Figure 2B are similar to those reported previously [3] with lower DP arabino-xyloglucan oligosaccharides eluting in the final preparative HPLC fraction (Figure 2C). Since cranberry rhamnogalacturonan oligosaccharides were partially separated from arabino-xyloglucan oligosaccharides with water elution from an ion-moderated partition preparative HPLC column, cranberry pectic and xyloglucan oligosaccharides do not appear to be covalently linked in A6.

### 3.3. Glycosyl-Linkage

The glycosyl- linkage analysis of three cranberry A6 fractions were similar, with various glucose linkages detected as the major residues in all the samples (Table 3). Aside from this, xylose and arabinose linkages were the major residues with galacturonic acid comprising a small amount of the total (Table 3). Rhamnogalacturonan I structures in Figure 2a are drawn with a 5-linked arabino-oligosaccharide or 3,6-branched-galacto-oligosaccharide side chains based on those abundant linkages (Table 3). However, the galacto-oligosaccharide side chain could also be exclusively 4-linked and the arabino-oligosaccharide could be branched at the 3-position based on the cranberry A6 linkages detected. The 2,4-Rha linkage was detected in only one of the cranberry A6 (C), which means that there was batch-to-batch variation in A6 rhamnogalacturonan oligosaccharide branching. The 3′-Apiose in the cranberry A6 C means that rhamnogalacturonan II was also present. Taken together, cranberry A6 contains significant amounts of arabino-xyloglucan (t-Ara, 2-Xyl, 4-Glc, 4,6-Glc) as well as a branched arabinan (t-Ara, 3-Ara, 5-Ara, 3,5-Ara and 2,3,5-Ara) with minor amounts of rhamnogalacturonan I and II.

### 3.4. Growth of Lactobacillus Plantarum BAA 793 on Prebiotics

**Figure 3:**
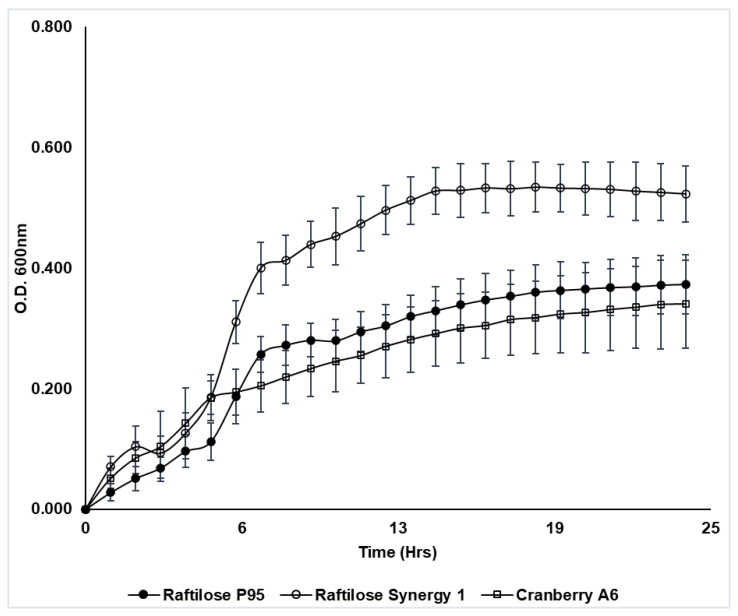
Growth of *Lactobacillus plantarum* ATCC BAA 793 in mMRS broth supplemented with Raftilose P95 (FOS, filled circles), Raftilose Synergy 1 (inulin, open circles) and cranberry A6 A (open squares). Growth was monitored by optical density (OD600) with data points representing the ΔOD (initial OD subtracted from all subsequent readings). Results are the average of three independent experiments ± SD.

Cranberry A6 was shown to support the growth of *Lactobacillus plantarum* ATCC BAA 793 (Figure 3), which agreed with previously reported results [30,40]. Results from our study showed that ATCC BAA 793 was also capable of fermenting FOS, with a final cell density comparable to growth on cranberry A6 A (Figure 3). This result differed from the previous study which reported that *L. plantarum* ATCC BAA 793 required the presence of a cranberry proanthocyanidin (PAC1) to stimulate FOS utilization [40], with the resulting cell density being significantly higher (OD_600_ 1.02) in the presence of PAC1. However, the mMRS used in the previous study did not contain acetate, which differed from the basal medium used in our study that included sodium acetate. More studies are required to determine if the presence of acetate affects *L. plantarum* ATCC BAA793 growth. Additionally, the same study reported that cranberry PAC1 could increase the growth of ATCC BAA 793 in the presence of glucose, human milk oligosaccharides (HMO) or cranberry xyloglucans, and in our study *L. plantarum* ATCC BAA793 utilized inulin (Synergy 1) as a sole carbon source in mMRS, reaching the highest culture density (ΔOD_600_ 0.52). Our results suggest that while cranberry xyloglucan can be utilized to support the growth of *L. plantarum* ATCC BAA 793, inulin may be a preferred carbon source resulting in higher biomass.

### 3.5. Growth of LAB on Cranberry A6

**Figure 4:**
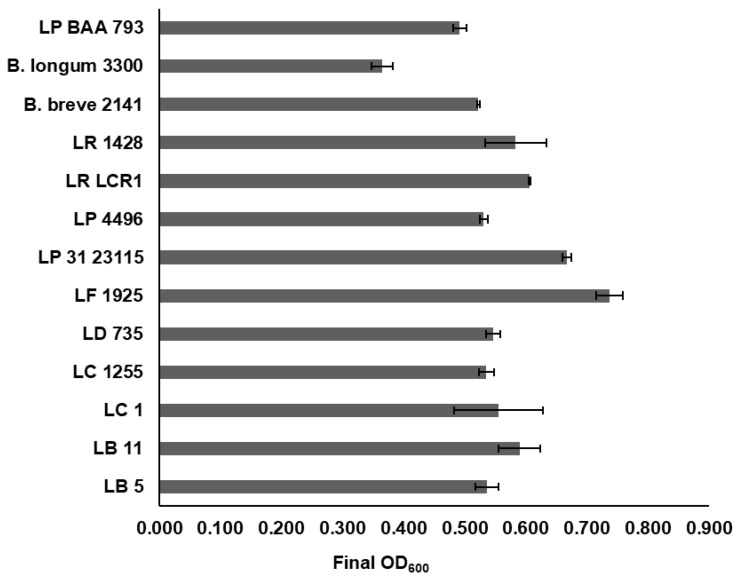
Final culture density (OD600) of selected *Lactobacillus* strains (*L. buchueri* (LB 5); *L. bulgaricus* (LB 11); *L. casei* ATCC 4646 (LC 1); *L. casei* B1255 (LC 1255); *L. delbreuckii* B735 (LD 735); *L. fermentum* B1925 (LF 1925); *L. paraplantarum* B23115 (LP 31 23115); *L. plantarum* B4496 (LP 4496); *L. rhamnosus* BLCR1 (LR LCR1); *L. reuteri* 1428 (LR 1428); *L. plantarum* ATCC BAA 793 (LP BAA 793)) and *Bifidobacterium* strains (*B. breve* 2141; *B. longum* subsp. *infantis* 3300) following growth in mMRS supplemented with 1% cranberry A6 A for 24 h at 32°. Results are the means from two independent experiments ± SD.

A screen using 86 *Lactobacillus* strains and two *Weissella* strains (Supplementary Table S2) identified ten which grew under anaerobic conditions in mMRS containing cranberry A6 A as a sole carbon source, reaching a final culture density (ΔOD) ≥ 0.50 after 24 h incubation at 32 °C (Figure 4). This group included *L. buchneri* LB5, *L. bulgaricus* LB11, *L. casei* strains ATCC 4646 and B1255, *L. delbreuckii* B735, *L. fermentum* B1925, *L. paraplantarum* B23115, *L. plantarum* B4496, *L. rhamnosus* BLCR1, and *L. reuteri* 1428, which all were observed to have a level of growth comparable or better than what was observed in a previous study for *L. plantarum* ATCC BAA 793 (Figure 4) [30], which reached a final culture density of 0.49. In addition to these ten strains, six other strains reached a final OD_600_ ≥ 0.40: *L. delbrueckii* B1658, *L. paracasei* B4564, *L. rhamnosus* strains B1937, B176 and B2327, and *L. sharpeae* B14855. Results from this study demonstrated that cranberry A6 utilization was strain-dependent, as several other strains of *Lactobacillus* species were unable to reach a final OD_600_ = 0.40, including: two *L. buchneri* (B1838, B1860), seven *L. bulgaricus* (LB1, LB6, LB12, LB15, LB21, YB1, B440), five *L. casei* (LC2, 393, B1922, B441, LC3), five *L. delbrueckii* (B443, B736, B1844, B1930, B4523), two *L. fermentum* (B585, B14171), five *L. plantarum* (14917, NCDO955, B1846, B1926, TSH076), and two *L. rhamnosus* (LGG, B1914) strains. A previous study in our laboratory looked at the growth of this panel of lactobacilli using commercial FOS and inulin as sole carbon sources and identified 12 which grew equally well on both substrates (OD_600_ > 1.0) [41]. When compared with the results from this study, three strains were identified as being able to utilize all three substrates: *L. casei* 4646, *L. paraplantarum* B23115 and *L. reuteri* 1428. In addition, *L. paracasei* 4564, which showed moderate growth on cranberry A6, was also capable of growing on both FOS and inulin (OD_600_ > 1.4) at 32 °C. Our previous study reported anaerobic conditions were not required for FOS or inulin utilization by *L. casei* 4646, and FOS utilization by *L. paracasei* B4564; however, results from our study showed all strains required anaerobic conditions for utilization of cranberry A6 (data not shown). Taken together, these results confirm that careful consideration needs to be given to the *Lactobacillus* strain, choice of prebiotic and environmental conditions when assessing oligosaccharide utilization, potentially for the development of a novel synbiotic.

*Bifidobacterium breve* 2141 and *Bifidobacterium longum* subsp. *infantis* 3300 were also investigated for their ability to grow on cranberry A6 as a sole carbon source, with *B. breve* 2141 shown to reach a final culture density (OD_600_) > 0.50 (Figure 4). *B. longum* subsp. *infantis* 3300 (ATCC 15697) was observed to reach a final OD_600_ < 0.40, which was not considered a significant level of growth in our study. In a previous study, it was also reported that *B. longum* subsp. *infantis* ATCC 15697 was unable to grow on cranberry xyloglucans [26]. The higher culture density observed in this study could have resulted from compositional differences between the cranberry A6 preparations used, or differing growth conditions which include the use of oxyrase to induce an anaerobic environment, and a lower incubation temperature of 32 °C. A previous study from our laboratory showed that some *Lactobacillus* strains utilized FOS or inulin better when grown at 32 °C instead of 37 °C, including strains *L. casei* 4646, *L. paracasei* B4564 and to a lesser extent *L. reuteri* 1428 which were identified as growing on cranberry A6 in this study [41]. Additionally, *L. rhamnosus* GG was reported to not utilize cranberry A6 in both the current study and the Ozcan et. al. [30] study, but in our study, it reached a final OD_600_ > 0.30 suggesting a higher level of background growth using our specific growth conditions (data not shown). Previously, *B. longum* subsp. *longum* (UD401) was the only *Bifidobacterium* strain reported to grow using cranberry xyloglucan as a sole carbon source, reaching a final OD_600_ = 0.15 [30], but this study showed that strains of *B. breve* may also be able to utilize this potential prebiotic.

### 3.6. Short Chain Fatty Acid Production by LAB Grown on Cranberry A6

**Figure 5:**
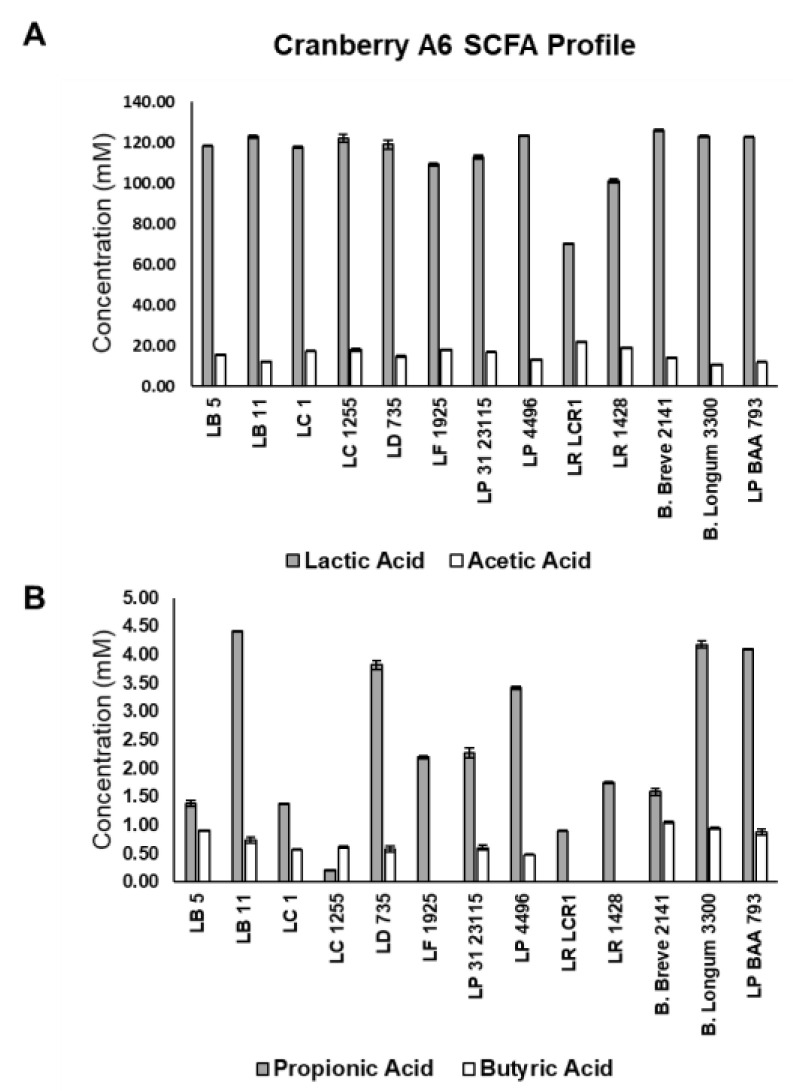
Short chain fatty acid production by *Lactobacillus* strains (*L. buchueri* (LB 5); *L. bulgaricus* (LB 11); *L. casei* ATCC 4646 (LC 1); *L. casei* B1255 (LC 1255); *L. delbreuckii* B735 (LD 735); *L. fermentum* B1925 (LF 1925); *L. paraplantarum* B23115 (LP 31 23115); *L. plantarum* B4496 (LP 4496); *L. rhamnosus* BLCR1 (LR LCR1); *L. reuteri* 1428 (LR 1428); *L. plantarum* ATCC BAA 793 (LP BAA 793)) and *Bifidobacterium* strains (*B. breve* 2141; *B. longum* subsp. *infantis* 3300) grown in mMRS supplemented with 1% cranberry A6 A. Production of lactic acid (shaded bars) and acetic acid (open bars) (**A**); and propionic acid (shaded bars) and butyric acid (open bars) (**B**) were determined by HPLC, and results are the means of three experiments ± SD.

Short chain fatty acid production was monitored for the 11 *Lactobacillus* strains and *Bifidobacterium breve* 2141, which showed optimal growth on cranberry A6 (Figure 5). All strains were shown to produce predominantly lactic acid (>70 mM), which agreed with previous results from our laboratory using FOS and inulin as the sole carbon source [41], but disagreed with a previous study that reported lactate production was <10 mM for *L. plantarum* ATCC BAA 793 and *B. longum* subsp. longum UD401 when grown on cranberry xyloglucans [30]. The same study reported acetate production was slightly higher than lactate in the presence of cranberry xyloglucans, but in our study the acetate concentration ranged from 11.9 to 22.1 mM for all strains, significantly lower than the amount of lactate produced in the other study. Acetate production by both bifidobacteria and lactobacilli has been reported under anaerobic and glucose limiting conditions [42,43], but the level of lactate production observed in our study suggested that the bacteria may not have been stressed under the current growth conditions when compared to other studies. Lactate being the primary organic acid produced also agreed with previous results from our laboratory when lactobacilli and bifidobacteria were grown in the presence of FOS and inulin as a sole carbon source [41].

Production of propionic and butyric acids was also monitored following growth on cranberry A6 (Figure 5B). All 11 lactobacilli and *B. breve* 2141 were shown to produce propionic acid (grey bars), and all but *L. fermentum* 1925, *L. rhamonosus* LRCLR1 and *L. reuteri* 1428 produced butyric acid (white bars). *L. plantarum* ATCC BAA 793 was one of the highest producers of both propionic (4.10 mM) and butyric (0.88 mM) acids, even though the other cultures appeared to grow better on cranberry A6 based on their final culture densities (Figure 4). This result suggests that optimal growth does not always correlate with optimal production of beneficial SCFAs, which agreed with the fecal fermentation results from our study that showed cranberry xyloglucans did not result in a population growth but increased the production of SCFAs (Figure 5, Table 4 and Table 5).

From the ten lactobacilli identified to grow on cranberry A6, *L. bulgaricus* LB11 (4.42 mM), *L. delbreuckii* subsp. *lactis* B735 (3.82 mM), and *L. plantarum* B4496 (3.42 mM) produced the highest concentrations of propionic acid; and *L. buchneri* (0.91 mM) and *L. bulgaricus* LB11 (0.74 mM) produced the most butyric acid. *B. breve* 2141 produced the highest concentration of butyric acid (1.05 mM) in our study, which was a similar concentration observed when grown on FOS (0.98 mM) or inulin (1.01 mM) [41]. However, *B. breve* 2141 was shown to produce a higher concentration of propionic acid (≥3.50 mM) on FOS or inulin, when compared to growth on cranberry A6 (1.58 mM). Additionally, *L. casei* 4646, *L. delbreuckii* B735 and *L. rhamnosus* LCR1 were previously reported to produce less propionic acid when grown on FOS (≤0.45 mM) or inulin (≤0.71 mM) as compared to cranberry xyloglucan, while butyric acid production was not detected using FOS or inulin. However, *L. reuteri* 1428 was reported to produce higher concentrations of propionic (≥2.74 mM) and butyric (≥1.22 mM) when FOS or inulin was provided as a sole carbon source [41]. Although lactobacilli and bifidobacterial are not considered primary producers of propionate or butyrate within the human intestinal microbiota [33], studies have reported their ability to produce these SCFAs [16,30,40,44]. The ability to produce propionate or butyrate could be viewed as a probiotic trait due to their reported health benefits [34,45,46].

Propionate has been reported to reduce fatty acid synthesis within hepatocytes and lower serum cholesterol levels; contribute to weight control as a regulator of satiety; and to induce apoptosis of colorectal carcinoma cell lines [47]. The concentration of propionate required to elicit these effects was reported to be between 9–20 mM for anticancer and antilipogenic effects [48,49], but significantly higher amounts to regulate satiety in humans (130–930 mM) [47]. The concentration of propionate produced by the *Lactobacillus* or *Bifidobacterium* strains in our study would not be sufficient to elicit this type of response but could potentially serve to raise the concentration produced by the indigenous microbiota if they could be for development of a synbiotic.

In humans, the intestinal microbiota produces less butyrate than acetate or propionate [1]; however, butyrate serves as a major energy source for colonocytes [50]. In addition, butyrate has been reported to inhibit histone deacetylase, which is responsible for its anticancer [51] and anti-inflammatory activities [52], and function as a signaling molecule via G protein-coupled receptors, which contribute to its immunomodulatory activities [53,54]. Butyrate has also been reported to enhance the barrier function of host epithelial cells [55] which contributes to its role in maintaining host gastrointestinal health. Unlike other health promoting activities associated with butyrate production, its effect on obesity remains controversial [56]. While butyrate has been reported to improve glucose homeostasis and relieve diet-induced obesity and insulin resistance in rodents [57,58], it has been linked to an increase in lipid synthesis [59] and overall higher concentrations of SCFAs have been reported in obese individuals when compared to lean individuals [60]. More work is needed to elucidate the role of butyrate on obesity, but the beneficial effects of butyrate continue to drive the search for probiotic bacteria capable of supplementing the concentration naturally produced by the indigenous microbiota.

The ten *Lactobacillus* strains identified for fermenting cranberry xyloglucans have not been investigated for potential probiotic activities, other than the production of SCFAs described within this study. *L. casei* 4646 is the most well-studied strain for its potential to contribute to the formation of dental caries since it was originally isolated from a carious lesion [61]. However, the Michalek et al. [61] study reported that in a rat model *L. casei* 4646 preferentially colonized the tongue and saliva, and when it was associated with the plaque microbiota at > 1% there was a reduction in *Streptococcus mutans*-induced dental caries. These results suggest that *L. casei* strains have potential as oral probiotics, with *L. casei* strain Shirota reported to prevent plaque-induced gingival inflammation [62]. Several other studies have also investigated the effects of *Lactobacillus* and *Bifidobacterium* strains, as well as strains of *Streptococcus thermophilus*, *Streptococcus salivarius*, *Lactococcus lactis* and *Enterococcus faecium* on oral health [63]. Additionally, several strains from the *L. casei* group, which consists of *L. casei*, *L. paracasei* and *L. rhamnosus*, have been studied for their ability to prevent allergic reactions, obesity, pathogen colonization and cancer; as well as modulate the immune system and enhance cognitive functions [64]. Several strains of each species have been studied for their health benefits, with *L. rhamnosus* GG being commercially available as a probiotic. Other species described in this study included *L. fermentum*, from which strains have been studied for their antimicrobial activity for food preservation and their cholesterol-lowering and anticancer potential [65]. Similarly, several studies have investigated the probiotic potential of *L. plantarum* and *L. paraplantarum* [66]; *L. delbreuckii* subsp. *bulgaricus* [67,68]; *L. buchneri* [69] and *L. reuteri* [70]. *B. breve* 2141 was also shown to grow on cranberry xyloglucan, and several strains of this species have been investigated for treatment of targeted pediatric diseases, including diarrhea, infant colic, celiac disease, obesity, allergies, and neurological disorders [71]. Taken together, these previous studies support the need for further studies to determine the probiotic potential of the *Lactobacillus* and *Bifidobacterium* strains identified as growing on cranberry xyloglucans to determine if they are feasible candidates for the development of synbiotics to improve human or animal health. Previously our laboratory demonstrated the survival and subsequent production of SCFAs by *L. reuteri* 1428 and *B. breve* 2141 within a synbiotic matrix containing FOS, inulin, or pectic-oligosaccharides [16], but additional studies are needed to determine if similar results would be obtained using cranberry xyloglucan A6 or pectic oligosaccharides.

### 3.7. Effect of Cranberry A6 on Fecal Microbiota Growth and SCFA Production

When fecal fermentations were performed using the preparative HPLC fraction 2 from cranberry A6 (extract 2), no significant growth was observed for any of the bacterial populations monitored (Table 4). For the same fecal samples, the presence of inulin resulted in a significant increase in bifidobacteria, lactobacilli and eubacteria (*p* < 0.05), which agreed with our previous study that reported FOS and citrus pectic-oligosaccharides induced *Bifidobacterium* growth within a mixed fecal fermentation [21]. The absence of prebiotic-induced growth did not agree with a previous study that reported whole cranberry powder, phenolic-enriched cranberry extract and phenolic-deficient cranberry extract broadly modulated bacterial growth within a human gut microbiome-derived community, with an increased abundance of *Bacteriodaceae* and a reduction in *Enterobacteriaceae* [72]. Another study supported the growth potential of xyloglucans, reporting that extracts from tucuma pulp led to larger shifts in bacterial fecal communities when compared with pectic polymers consisting of homogalacturonans and rhamnogalacturonans [35]. Unfortunately, crude cranberry pomace did not dissolve in the basal medium, which prevented its use in Flow-FISH analysis to monitor bacterial growth, and further analysis of the cranberry preparative HPLC fractions identified trace amounts of silver, which may have inhibited growth of the fecal microbiota. Another explanation for these results may be that cranberry arabino-xyloglucan is not as active as a prebiotic compared to cranberry rhamnogalacturonan I, which was enriched in our preparative HPLC fraction 1 (Figure 2). Onumpai et al. [24] reported that arabinan and galactan oligosaccharides had optimal prebiotic activity for pectic oligosaccharides analyzed in mixed fecal fermentations using FISH analysis. Previous results supporting the prebiotic activity of cranberry xyloglucan used fractions that may have also contained small amounts of arabino-oligosaccharides that are very active fermentable carbohydrates. These results emphasize the need for additional studies to determine why the cranberry preparative HPLC fraction 2 was unable to elicit changes in bacterial growth.

Short chain fatty acid production was also monitored in fecal batch cultures, and the presence of cranberry pomace and preparative HPLC fraction 2 from cranberry A6 resulted in a significantly increased abundance of propionate and butyrate (*p* < 0.05) compared to the negative control at 24 h (Table 5). Additionally, SCFA production in the presence of crude cranberry pomace elicited similar results to what was observed when inulin was used as a prebiotic. The lower abundance of both acetate and butyrate observed when the cranberry preparative HPLC fraction 2 was used compared to cranberry pomace, could also be due to the lack of *Lactobacillus* growth, which would agree with our observations from in vitro studies using specific *Lactobacillus* strains (Table 1). Since crude cranberry pomace could not be used for Flow-FISH analysis of bacterial growth studies, it is not possible to determine if increases in SCFAs correlated with increases in bacterial populations. Although the lack of bacterial growth may contribute to the lower abundance of SCFAs, results from this study do agree with a previous study which reported low amounts of dietary fiber can increase the metabolic activity of the colonic microbiota leading to the production of SCFAs without changes to the microbial composition [73].

## 4. Conclusions

The carbohydrate composition and structure of cranberry pectic oligosaccharides was described as rhamnogalacturonan I structures which were methyl-esterified, acetylated with arabinogalacto-oligosaccharide side chains and a 4,5-unsaturated function at their non-reducing ends. Since arabinose-rich pectic oligosaccharides were previously reported with prebiotic activity, this suggested that the cranberry A6 fraction could also stimulate the growth and short-chain fatty acid production of health-promoting bacteria. Of the 86 *Lactobacillus* strains screened for growth on cranberry A6 as the sole carbon source in single culture studies, ten grew well at a final culture density (ΔOD) ≥ 0.50 after 24 h incubation at 32 °C. All of these ten *Lactobacillus* strains produced lactic, acetic and propionic acids, and all but three strains produced low levels of butyric acid. This was the first report that the *Lactobacillus* ability to metabolize cranberry oligosaccharides is strain specific, and this is the first study to demonstrate that several *Lactobacillus* species, other than *L. plantarum*, can utilize this potential prebiotic. In mixed culture fecal fermentations using FISH analysis with 16s rRNA probes, no bacterial class had significant growth when the cranberry preparative HPLC fraction enriched in arabino-xyloglucan was included yet acetate, propionate and butyrate increased in these fecal fermentations. The even higher level of SCFAs produced when crude cranberry pomace was included in fecal fermentations suggests that other cranberry compounds may contribute to its microbial modulating effect. More research is necessary to completely determine the cranberry prebiotic structure/function relationships.

## Figures and Tables

**Table 1:** Molecular characteristics of cranberry A6 fraction.

	%Mass Fraction ^a^	*M_w_*/Mn	M_z_/M_n_	*M_w_* (kDa)	*η_w_* (dL/g)	Rhzv (nm)	M-H
A6	97.8 (0.2)	1.03 (0.03)	1.07 (0.1)	1.48 (0.3)	0.025 (0.001)	1.0 (0.1)	0.260 (0.1)
A6 ^b^	103.0 (1.0)	1.17 (0.04)	1.64 (0.2)	2.75 (0.2)	0.044 (0.002)	1.4 (0.01)	0.701 (0.02)

^a^ Percentage (%) of an integrated peak area over the total of all eluted peak areas. ^b^ Hotchkiss et al. [3]. Standard deviation is shown in parentheses (*n* = 3).

**Table 2:** Monosaccharide composition (mole %).

Sample	Glc	Ara	Gal	Xyl	Rha	Fuc	GalA	GlcA
A6 A	40.9	16.1	3.7	30.4	0.5	0.1	7.9	0.5
A6 B	38. 8	17.7	0.9	6.8	31.9	0.4	1.0	2.5
Prep HPLC fraction 1	18.7	31.0	8.9	13.7	6.8	1.5	17.6	2.0
Prep HPLC fraction 2	37.1	26.4	4.0	30.9	ND	0.2	1.3	0.0
Prep HPLC fraction 3	40.1	14.5	7.6	35.3	ND	0.6	1.6	0.3

Glucose (Glc), arabinose (Ara), galactose (Gal), xylose (Xyl), rhamnose (Rha), fucose (Fuc), galacturonic acid (GalA) and glucuronic acid (GlcA) mole % values determined by HPAEC-PAD following methanolysis. A6 A and A6 B are different batches produced in different years. ND = not detected.

**Table 3:** Glycosyl linkage analysis of cranberry A6 fractions.

Linkage	A6 A	A6 B	A6 C
t-Ara*f*	5.7	5.5	7.7
t-Fuc*p*	0.1	0.3	0.2
t-Ara*p*	0.1	0.3	0.1
t-Xyl*p*	3.3	2.1	3.8
2-Rha*p*	0.1	0.4	0.7
3-Rha*p*	-	0.2	0.2
t-Man*p*	0.1	-	-
t-Glc*p*	15.5	26.9	8.1
3-Ara*f*	0.7	1.7	2.5
t-Gal*f*A	-	-	0.6
t-Gal*p*	2.5	1.6	2.8
t-Gal*p*A	0.2	0.2	0.7
4-Ara*p* or 5-Ara*f*	4.0	8.2	12.0
3’-Apiose	-	-	0.1
2-Xyl*p*	9.9	4.5	7.0
4-Xyl*p*	1.2	0.9	1.0
3,4-Fuc*p*	0.1	0.1	0.2
3-Glc*p*	0.3	1.3	-
2,4-Rha*p*	-	-	0.5
2-Glc*p*	0.2	0.4	0.1
2-Glc*p*A	-	-	0.1
3-Gal*p*	0.1	-	0.2
4-Man*p*	0.4	0.2	0.5
3,4-Ara*p* or 3,5-Ara*f*	0.3	1.3	2.5
6-Glc*p*	5.9	11.7	5.8
4-Gal*p*	0.1	0.1	0.3
4-Gal*p*A	0.4	0.2	1.7
4-Glc*p*	20.0	17.7	17.4
2,4-Xyl*p*	0.1	0.1	0.1
6-Gal*p*	0.1	0.1	0.3
2,3,4-Ara*p* or 2,3,5-Ara*f*	0.1	0.2	0.9
3,4-Glc*p*	0.2	0.2	0.3
2,4-Glc*p*	0.5	0.3	0.4
2,4-Glc*p*A	-	-	0.1
3,6-Glc*p*	-	-	0.1
4,6-Glc*p*	27.7	13.2	20.6
3,6-Gal*p*	0.1	-	0.1

Glycosyl-linkage deduced from GC/MS analysis of per-O-methylated alditol acetates. Glycosyl-linkage position (numbers), apiose (Api), arabinose (Ara), fructose (Fru), galactose (Gal), galacturonic acid (GalA), glucose (Glc), glucuronic acid (GlcA), fucose (Fuc), mannose (Man), rhamnose (Rha), xylose (Xyl), furanose (f), pyranose (p), terminal (t) and not detected (-). Relative peak area percentages (%) three cranberry batches (A6 A, A6 B, A6 C) were produced in different years.

**Table 4:** Mean bacterial populations (Log CFU mL^−1^) of three donors in pH-controlled miniature fecal batch cultures supplemented with cranberry preparative HPLC fraction 2 (cranberry extract 2) at 0, 24 and 48 h.

Probe	Time (h)	Negative Control	Inulin	Cranberry Extract 2
EUB	0	7.25 (0.50) ^a^	7.25 (0.47) ^a^	7.25 (0.40) ^a^
	24	7.33 (0.61) ^a^	7.73 (0.22) ^a^*	7.55 (0.41) ^a^
	48	7.06 (0.42) ^a^	7.69 (0.18) ^ab^	7.35 (0.20) ^ab^
BIF	0	5.91 (0.85) ^a^	5.88 (0.85) ^a^	5.87 (0.67) ^a^
	24	6.01 (1.01) ^a^	7.03 (0.59) ^a^	6.28 (0.47) ^a^
	48	5.86 (0.76) ^a^	7.32 (0.26) ^c^*	5.93 (0.22) ^ab^
LAB	0	4.99 (0.51) ^a^	4.95 (0.34) ^a^	4.76 (0.73) ^a^
	24	5.30 (0.72) ^a^	5.94 (0.21) ^a^*	5.57 (0.61) ^a^
	48	5.06 (0.85) ^a^	5.90 (0.38) ^a^*	5.65 (0.28) ^a^
BAC	0	5.85 (0.16) ^a^	5.87 (0.20) ^a^	5.81 (0.34) ^a^
	24	6.35 (0.43) ^a^	6.40 (0.39) ^a^	6.28 (0.55) ^a^
	48	5.92 (0.46) ^a^	6.21 (0.26) ^a^	5.98 (0.41) ^a^
EREC	0	6.84 (0.45) ^a^	6.82 (0.47) ^a^	6.85 (0.33) ^a^
	24	6.69 (0.47) ^a^	7.00 (0.22) ^a^	6.98 (0.64) ^a^
	48	6.02 (0.57) ^a^	6.56 (0.20) ^ab^	6.81 (0.22) ^ab^
RREC	0	6.07 (0.47) ^a^	6.17 (0.50) ^a^	5.91 (0.50) ^a^
	24	5.86 (0.63) ^a^	6.59 (0.25) ^a^	6.11 (0.81) ^a^
	48	5.10 (0.94) ^a^	5.95 (0.41) ^a^	5.96 (0.43) ^a^
ATO	0	5.37 (0.33) ^a^	5.42 (0.35) ^a^	5.38 (0.54) ^a^
	24	5.91 (0.64) ^a^	6.21 (0.46) ^a^	5.48 (0.81) ^a^
	48	5.64 (0.23) ^a^	6.02 (0.54) ^a^	5.56 (0.44) ^a^
PRO	0	5.56 (0.55) ^a^	5.67 (0.41) ^a^	5.62 (0.59) ^a^
	24	6.04 (0.89) ^a^	6.18 (0.38) ^a^	5.91 (0.68) ^a^
	48	5.56 (0.45) ^a^	5.97 (0.47) ^a^	5.78 (0.47) ^a^
FPRAU	0	6.54 (0.60) ^a^	6.57 (0.49) ^a^	6.61 (0.43) ^a^
	24	6.38 (0.86) ^a^	6.90 (0.40) ^a^	7.00 (0.14) ^a^
	48	5.85 (0.99) ^a^	6.82 (0.39) ^a^	6.70 (0.54) ^a^
DSV	0	6.27 (0.54) ^a^	6.30 (0.37) ^a^	6.37 (0.41) ^a^
	24	5.87 (0.84) ^a^	6.04 (0.37) ^a^	5.65 (1.01) ^a^
	48	5.22 (0.65) ^a^	6.11 (0.41) ^ab^	5.49 (0.37) ^ab^
CHIS	0	4.86 (0.72) ^a^	4.61 (0.71) ^a^	4.41 (0.93) ^a^
	24	5.02 (1.00) ^a^	5.40 (0.45) ^a^	5.91 (0.31) ^a±^
	48	4.87 (0.94) ^a^	5.73 (0.57) ^a^	5.74 (0.45) ^a±^

Standard deviation is shown in parentheses (*n* = 3). Significant differences (*p* < 0.05) among substrates are indicated with different letters in a same row. * Significant differences from 0 h, *p* ≤ 0.05; ^±^ non-significant trend, *p* ≤ 0.1.

**Table 5:** SCFA production in pH-controlled miniature fecal batch cultures supplemented with cranberry pomace or cranberry preparative HPLC fraction 2 (extract 2) at 0, 24 and 48 h.

		Negative Control		Inulin		Cranberry Pomace		Cranberry Extract 2	
SCFA	Time								
Acetate	t0	10.55	(3.29)	12.62	(3.15)	7.67	(1.57)	4.49	(0.82)
	t24	8.98	(1.38)	21.93	(2.12) ^a^	19.57	(2.64) ^±^	18.15	(1.78) ^±^
	t48	14.23	(0.87)	20.11	(2.14) ^a^	20.73	(2.58) ^±^	17.12	(1.27) ^±^
Propionate	t0	3.37	(3.29)	2.92	(3.15)	1.62	(1.57)	0.76	(0.82)
	t24	2.31	(1.38)	5.76	(2.12)	7.53	(2.64) ^b^	7.73	(1.78) *^a^
	t48	4.27	(0.87)	6.43	(2.14)	8.41	(2.58) ^±^	9.40	(1.27) *^a^
Butyrate	t0	3.90	(2.54)	3.39	(2.17)	2.34	(1.02)	0.36	(0.62)
	t24	4.54	(3.96)	26.23	(15.58) ^±^	18.39	(3.55) *^a^	17.01	(6.51) *^a^
	t48	6.52	(4.69)	25.97	(12.47) ^±^	22.57	(3.02) **	15.31	(9.29) ^±^
Valerate	t0	0.00	(0.00)	0.00	(0.00)	0.00	(0.00)	0.00	(0.00)
	t24	2.62	(3.43)	0.00	(0.00)	2.70	(2.53)	0.00	(0.00)
	t48	2.88	(2.96)	0.47	(0.81)	4.52	(0.91) *	0.00	(0.00)
Isobutyrate	t0	0.00	(0.00)	0.00	(0.00)	0.00	(0.00)	0.00	(0.00)
	t24	2.26	(1.32) ^±^	0.00	(0.00) ^a^	1.26	(2.18)	1.21	(2.10)
	t48	2.12	(1.68)	0.95	(1.64)	1.72	(2.98)	1.79	(2.46)
Isovalerate	t0	0.00	(0.00)	0.00	(0.00)	0.00	(0.00)	0.00	(0.00)
	t24	2.74	(0.85) *	0.00	(0.00) ^a^	1.09	(1.89)	1.17	(1.30)
	t48	2.25	(1.04)	0.00	(0.00)	2.47	(1.58) *	0.85	(1.48)

Short chain fatty acid concentrations (mM) during fermentations. Standard deviation is shown in parentheses (*n* = 3). Significant differences to control at same time point denoted by ^a^ (*p* < 0.05), ^b^ *p* < 0.01). * Significant differences from value of same substrate at 0 h, *p* ≤ 0.05; ** significant differences from value 0 h *p* < 0.01; ^±^ non-significant trend, *p* ≤ 0.1.

## Data Availability

The data generated in this study will be available in Food Data Central.

## Supplementary Materials

The following supporting information can be downloaded at: https://www.mdpi.com/article/10.3390/microorganisms10071346/s1, Table S1: MALDI-TOF MS Analysis of Cranberry Oligosaccharides in Preparative HPLC Fractions; Table S2: Lactobacillus and Bifidobacterium species in used in this study.

## Statement

Mention of trade names or commercial products in this publication is solely for the purpose of providing specific information and does not imply recommendation or endorsement by the U.S. Department of Agriculture. USDA is an equal opportunity provider and employer.
