# Supplementary material for: Cranberry Arabino-Xyloglucan and Pectic Oligosaccharides Induce Lactobacillus Growth and Short-Chain Fatty Acid Production
Source: Microorganisms. 2022 Jul 3;10(7):1346. doi: 10.3390/microorganisms10071346 (PMC9319371; doi:10.3390/microorganisms10071346)
Supplement: Supplementary file 1 [file microorganisms-10-01346-s001.zip › microorganisms-1776152-supplementary.pdf]

microorganisms

MDPI

Article

Cranberry Arabino-Xyloglucan and Pectic Oligosaccharides Induce *Lactobacillus* Growth and Short-Chain Fatty Acid Production

Arland T. Hotchkiss, Jr. [1,*,†], John A. Renye, Jr. [1,†], Andre K. White [1], Alberto Nunez [1], Giselle K. P. Guron [1], Hoa Chau [1], Stefanie Simon [2], Carlos Poveda [3], Gemma Walton [3], Robert Rastall [3] and Christina Khoo [4]

[1] Dairy & Functional Foods Research Unit, Agricultural Research Service, United States Department of Agriculture, Wyndmoor, PA 19038, USA; john.renye@usda.gov (J.A.R.J.); andre.white@usda.gov (A.K.W.); alberto.nunez@usda.gov (A.N.); giselle.guron@usda.gov (G.K.P.G.); rose.chau@usda.gov (H.C.)

[2] Sustainable Biofuels and Co-Products Research Unit, Agricultural Research Service, U.S. Department of Agriculture, Wyndmoor, PA 19038, USA; stefanie.simon@usda.gov

[3] Department of Food and Nutritional Sciences, The University of Reading, Harry Nursten Building, Pepper Lane, Whiteknights, Reading RG6 6DZ, UK; c.g.povedaturrado@reading.ac.uk (C.P.); g.e.walton@reading.ac.uk (G.W.); r.a.rastall@reading.ac.uk (R.R.)

[4] Ocean Spray Cranberries, Inc., One Ocean Spray Drive, Lakeville-Middleboro, MA 02349, USA; ckhoo@oceanspray.com

* Correspondence: arland.hotchkiss@usda.gov; Tel.: +1-215-233-6448

† These authors contributed equally to this work.

Supplementary Materials:

Table S1. MALDI-TOF MS Analysis of Cranberry Oligosaccharides in Preparative HPLC Fractions.

m/z (Na+)	Structure
761	Pent3Hex2
769	Pent3dHexuHexAMe2
785	Pent2dHexuHexAMe
789	Pent2dHexHexHexA
791	Pent2Hex3
815	PentdHexHexHexAuHexA
829	PentdHexHexHexAuHexAMe
831	Pent2dHexHexHexAAc
833	Pent6 or Pent2Hex3Ac
843	PentdHexHexHexAuHexAMe2
855	Pent2dHexHexAuHexAAcMe2
857	PentdHexHexHexAuHexAAc
923	Pent3Hex3
945	Pent3dHexHexAuHexAMe2
953	Pent2Hex4
959	Pent3dHexHexAuHexAAc
965	Pent7 or Pent3Hex3Ac
973	Pent3dHexHexAuHexAAcMe
987	Pent3dHexHexAuHexAAcMe2
989	Pent2dHexHexHexAuHexAAc
995	Pent6Hex

m/z(Na+)	Structure
1315	PentdHexHex3HexA2uHexA
1333	Pent6Hex2HexA
1341	Pent2dHexHexHexA3uHexAAc
1345	dHexHex4HexA2uHexA
1357	Pent5dHexHexHexAuHexAMe
1363	Pent5Hex3HexA
1375	PentdHexHex3HexA3Ac
1379	Pent4Hex5
1393	Pent4Hex5Me
1423	Pent3Hex6Me
1477	PentdHexHex4HexA2uHexA
1495	Pent6Hex3HexA
1507	dHexHex5HexA2uHexA
1511	Pent5Hex5
1525	Pent5Hex4HexA
1533	Pent5dHexHex2HexAuHexAMe2
1541	Pent2dHex3HexA3uHexAAc2Me2
1555	Pent4Hex6Me
1571	PentdHex3HexHexA3uHexAAc2Me2
1579	Pent3dHexHex3HexA2uHex
1669	dHexHex6HexA2uHexA
1673	Pent3dHex3HexA3uHexAAc2Me2

1003	Pent2dHexHexHexAuHex-AAcMe
1009	Pent6HexA
1017	Pent2dHexHexHexAuHex-AAcMe2
1055	Pent4Hex3
1069	Pent4Hex2HexA
1085	Pent3Hex4
1097	Pent8 or Pent4Hex3Ac
1099	Pent3Hex4Me
1123	Pent2dHexHexHexA2uHexA
1135	Pent3dHexHexHexAuHex-AAcMe
1153	PentdHexHex2HexA2uHexA
1149	Pent3dHexHexHexAuHex-AAcMe2
1171	Pent6HexHexA
1183	dHexHex3HexA2uHexA
1201	Pent5Hex2HexA
1211	Pent4dHexHexHexAuHexA
1217	Pent4Hex4
1229	Pent9
1247	Pent3Hex5
1253	Pent4dHexHexHexAuHexAAc
1261	Pent3Hex5Me
1281	Pent4dHexHexHexAuHex-AAcMe2
1313	Pent2dHexHex3HexAuHexAAc
1703	Pent2dHex3HexHexA3uHexAAc2Me2
1805	Pent2dHex3Hex2HexA3uHexAAc2Me2
1835	Pent4dHex3HexA3uHexAAc2Me2
1865	Pent3dHex3HexHexA3uHexAAc2 Me2
1937	Pent5dHex3HexA3uHexAAc2Me2
1967	Pent4dHex3HexHexA3uHexAAc2Me2
1997	Pent3dHex3Hex2HexA3uHexAAc2Me2
2069	Pent6dHex3HexA3uHexAAc2Me2
2099	Pent5dHex3HexHexA3uHexAAc2 Me2
2129	Pent4dHex3Hex2HexA3uHexAAc2Me2
2159	Pent3dHex3Hex3HexA3uHexAAc2Me2
2231	Pent6dHex3HexHexA3uHexAAc2Me2
2261	Pent5dHex3Hex2HexA3uHexAAc2Me2
2291	Pent4dHex3Hex3HexA3uHexAAc2Me2
2321	Pent3dHex3Hex4HexA3uHexAAc2Me2
2393	Pent6dHex3Hex2HexA3uHexAAc2Me2
2423	Pent5dHex3Hex3HexA3uHexAAc2Me2
2453	Pent4dHex3Hex4HexA3uHexAAc2Me2
2525	Pent7dHex3Hex2HexA3uHexAAc2Me2
2555	Pent6dHex3Hex3HexA3uHexAAc2Me2
2585	Pent5dHex3Hex4HexA3uHexAAc2Me2
2717	Pent6dHex3Hex4HexA3uHexAAc2Me2

Table S2. *Lactobacillus* and *Bifidobacterium* species in used in this study.

Bacteria	Source[1]
L. acidophilus LA1	DFF
L. acidophilus LA 6	DFF
L. acidophilus LA 8	DFF
L. acidophilus LA 10	DFF
L. acidophilus LA 15	DFF
L. acidophilus LA 30	DFF
L. acidophilus B1912	NRRL
L. acidophilus RP32	DFF
L. amylophilus B4437	NRRL
L. amylophilus B4481	NRRL
L. amylovorus B4540	NRRL
L. amylovorus B4548	NRRL
L. animalis B14176	NRRL
L. animalis B14177	NRRL
L. arabinosis B787	NRRL
L. brevis B4527	NRRL
L. brevis B1127	NRRL
L. brevis 3057	DFF
L. brevis 1836	DFF
L. buchneri LB5	DFF
L. buchneri B1838	NRRL
L. buchneri B1860	NRRL

L. bulgaricus LB1	DFF
L. bulgaricus LB6	DFF
L. bulgaricus LB11	DFF
L. bulgaricus LB12	DFF
L. bulgaricus LB15	DFF
L. bulgaricus LB21	DFF
L. bulgaricus YB1	DFF
L. bulgaricus B440	NRRL
L. casei 4646	*ATCC*
L. casei LC2	DFF
L. casei 393	ATCC
L. casei B1922	NRRL
L. casei B441	NRRL
L. casei LC3	DFF
L. casei B1255	NRRL
L. rhamnosus EV2	DFF
L. rhamnosus B442	NRRL
L. coryniformis ssp. *torquens* B4390	NRRL
L. curvatus B4562	NRRL
L. delbrueckii ssp. *lactis* B735	NRRL
L. delbrueckii B443	NRRL
*L. delbrueckii B*1658	NRRL
L. delbrueckii ssp. *lactis* B736	NRRL
L. delbrueckii ssp. *lactis* B1844	NRRL
L. delbrueckii ssp. *lactis* B1930	NRRL
L. delbrueckii ssp. *lactis* B4523	NRRL
L. fermentum B4525	NRRL
L. farciminis LF 25 (B4566)	NRRL
L. fermentum B585	NRRL
L. fermentum B1925	NRRL
L. fermentum B14171	NRRL
L. fructosus 2041	DFF
L. gasseri 1912	DFF
L. gasseri 4240	DFF
L. gasseri 14175	DFF
L. helveticus B4526	NRRL
L. helveticus EV1	DFF
L. helveticus B1842	NRRL
L. helveticus B1935	NRRL
L. helveticus B1942	NRRL
L. helveticus B1929	NRRL
L. lactis FARR	OSU
L. mali B4563	NRRL
L. paracasei B4564	NRRL
L. paraplantarum B23115	NRRL
L. plantarum 14917	*ATCC*
L. plantarum NCDO955	DFF
L. plantarum B4496	NRRL
L. plantarum B1846	NRRL
L. plantarum B1926	NRRL
L. plantarum TSH076	NRRL
L. plantarum BAA793	ATCC
L. reuteri 23272	ATCC
L. rhamnosus GG	ATCC
L. rhamnosus B176	NRRL
L. rhamnosus B1914	*NRRL*
L. rhamnosus B1937	NRRL
L. rhamnosus BLCR1	DFF
L. ruminus 14583	DFF
L. salivarius ssp. *salivarius* B1949	NRRL

L. salivarius ssp. *salicinus* B1950	NRRL
L. sharpeae B14855	NRRL
L. (Weissella) viridescens B1951	NRRL
L. (Weissella) confusa B1064	NRRL
L. acidophilus 1426	Luchansky
L. reuteri 1428	Luchansky
Bifidobacterium breve 2141 (ATCC 15698)	Luchansky
Bifidobacterium longum subsp. infantis 3300 (ATCC 15697)	Luchansky
